# Prediction of SARS-CoV-2-Related Lung Inflammation Spreading by V:ERITAS (Vanvitelli Early Recognition of Inflamed Thoracic Areas Spreading)

**DOI:** 10.3390/jcm11092434

**Published:** 2022-04-26

**Authors:** Ciro Romano, Domenico Cozzolino, Giovanna Cuomo, Marianna Abitabile, Caterina Carusone, Francesca Cinone, Francesco Nappo, Riccardo Nevola, Ausilia Sellitto, Annamaria Auricchio, Francesca Cardella, Giovanni Del Sorbo, Eva Lieto, Gennaro Galizia, Luigi Elio Adinolfi, Aldo Marrone, Luca Rinaldi

**Affiliations:** 1 COVID Center, Division of Internal Medicine, Department of Advanced Medical and Surgical Sciences, “Luigi Vanvitelli” University of Campania, 80131 Naples, Italy; domenico.cozzolino@unicampania.it (D.C.); abitabilemarianna@gmail.com (M.A.); katiacarusone@libero.it (C.C.); francesca_cinone@yahoo.it (F.C.); marcello.nappo@libero.it (F.N.); riccardo.nevola@unicampania.it (R.N.); cristianasellitto@gmail.com (A.S.); luigielio.adinolfi@unicampania.it (L.E.A.); aldo.marrone@unicampania.it (A.M.); 2 Rheumatology Unit, Department of Precision Medicine, “Luigi Vanvitelli” University of Campania, 80131 Naples, Italy; giovanna.cuomo@unicampania.it; 3 Division of Gastrointestinal Tract Surgical Oncology, Department of Translational Medical Sciences, “Luigi Vanvitelli” University of Campania, 80138 Naples, Italy; annamaria.auricchio@unicampania.it (A.A.); francesca.cardella@unicampania.it (F.C.); giovanni.delsorbo@unicampania.it (G.D.S.); eva.lieto@unicampania.it (E.L.); gennaro.galizia@unicampania.it (G.G.)

**Keywords:** COVID-19, pneumonia, risk prediction, HFNC, CPAP, OTI

## Abstract

*Background* Coronavirus disease 2019 (COVID-19) can be complicated by interstitial pneumonia, possibly leading to severe acute respiratory failure and death. Because of variable evolution ranging from asymptomatic cases to the need for invasive ventilation, COVID-19 outcomes cannot be precisely predicted on admission. The aim of this study was to provide a simple tool able to predict the outcome of COVID-19 pneumonia on admission to a low-intensity ward in order to better plan management strategies for these patients. *Methods* The clinical records of 123 eligible patients were reviewed. The following variables were analyzed on admission: chest computed tomography severity score (CTSS), PaO_2_/FiO_2_ ratio, lactate dehydrogenase (LDH), neutrophil to lymphocyte ratio (NLR), lymphocyte to monocyte ratio, C-reactive protein (CRP), fibrinogen, D-dimer, aspartate aminotransferase (AST), alanine aminotransferase, alkaline phosphatase, and albumin. The main outcome was the intensity of respiratory support (RS). To simplify the statistical analysis, patients were split into two main groups: those requiring no or low/moderate oxygen support (group 1); and those needing subintensive/intensive RS up to mechanical ventilation (group 2). *Results* The RS intensity was significantly associated with higher CTSS and NLR scores; lower PaO_2_/FiO_2_ ratios; and higher serum levels of LDH, CRP, D-dimer, and AST. After multivariate logistic regression and ROC curve analysis, CTSS and LDH were shown to be the best predictors of respiratory function worsening. *Conclusions* Two easy-to-obtain parameters (CTSS and LDH) were able to reliably predict a worse evolution of COVID-19 pneumonia with values of >7 and >328 U/L, respectively.

## 1. Introduction

Coronavirus disease of 2019 (COVID-19), caused by the recently identified severe acute respiratory syndrome coronavirus 2 (SARS-CoV-2), can be complicated by interstitial pneumonia, possibly leading to severe acute respiratory failure and death [1,2]. Although individual patient characteristics and comorbidities, such as advanced age, male sex, obesity, hypertension, diabetes, and/or coronary heart disease, have all been shown to be significantly associated with mortality in COVID-19 patients [3,4], the early identification of high-risk COVID-19 patients may still be challenging. Recent studies have thus investigated the predictive ability of several laboratory tests in order to quickly identify COVID-19 patients prone to severe respiratory involvement, including C-reactive protein (CRP), ferritin, and D-dimer, among others [1,5,6], and several groups have proposed predictive risk scores based on their own experience with COVID-19 patients. However, none of these have been successfully implemented in clinical practice as of yet; moreover, these scores are sometimes cumbersome, and a dedicated calculator may be necessary for those including a large number of variables [7,8,9]. The aim of this study was thus to identify a novel, simple approach to use for the early identification of COVID-19 patients prone to developing severe manifestations of interstitial pneumonia. To this end, we primarily focused on CT scan data to assess the extent of lung involvement, along with serum determination of LDH, as a surrogate lab test measure of lung damage severity. Other previously investigated variables were also included in the statistical analysis to sort out their possible contribution to the predictive model.

## 2. Patients and Methods

### 2.1. Patient Chart Review

We retrospectively reviewed the medical records of all consecutive COVID-19-positive subjects admitted to our medical division from 13 December 2020 to 17 May 2021. All patients were initially evaluated in the emergency room of nearby hospitals and then immediately transferred to our medical division (same-day admission). Initial management strategies were thus the same for all patients. Patients with severe respiratory distress already requiring intensive treatments were not suitable for treatment in our division and were referred to the appropriate wards. To be eligible for the study, patients needed to have undergone routine laboratory tests, evaluation of PaO_2_/FiO_2_ (P/F) ratio, and chest computed tomography (CT) with CT severity score (CTSS) assessment according to Chung et al. [10]. Briefly, each of the five lung lobes was assessed for the degree of involvement and classified as none (0%), minimal (1–25%), mild (26–50%), moderate (51–75%), or severe (76–100%). No involvement corresponded to a lobe score of 0, minimal involvement to a lobe score of 1, mild involvement to a lobe score of 2, moderate involvement to a lobe score of 3, and severe involvement to a lobe score of 4. An overall lung severity score was obtained by summing the five lobe scores (range of possible scores: 0–20). The chest CT scan was performed on the same day of hospital admission by a dedicated radiologist with expertise in COVID-19 pneumonia. Laboratory tests used to predict outcomes in this study included lactate dehydrogenase (LDH), neutrophil to lymphocyte ratio (NLR), lymphocyte to monocyte ratio (LMR), C-reactive protein (CRP), fibrinogen, D-dimer, aspartate aminotransferase (AST), alanine aminotransferase (ALT), alkaline phosphatase (ALP), and albumin. Values obtained on admission at the first blood draw were used for statistical analyses.

### 2.2. Definition of Outcome and Group Definitions

The main outcome was the intensity of respiratory support (RS). Oxygen was administered according to suggested national guidelines [11]. To simplify the statistical analysis, patients were split into two main groups according to the respiratory outcome: (i) those remaining in ambient air respiration plus those requiring oxygen delivered through either nasal or high-flow nasal cannulas (HFNC) (group 1, no or mild/moderate RS) and (ii) those needing continuous positive airway pressure (CPAP), non-invasive ventilation (NIV), or orotracheal intubation (OTI) with mechanical ventilation (group 2, intensive RS). This categorization of RS groups was chosen on the basis of the maximum level of care that should be delivered in a non-intensive setting (e.g., an internal medicine ward, group 1 patients) or whether it would be more appropriately delivered in an intensive setting (sub-intensive/critical care units, group 2 patients). Standard treatment comprised dexamethasone, low-molecular-weight heparin, and supportive measures, as appropriate [12,13]; comorbidities were treated according to specific established guidelines. Patients undergoing severe worsening of respiratory function were offered treatment with tocilizumab as a last-resort therapy for cytokine-mediated hyperinflammation syndrome [14]. As tocilizumab therapy was implemented after worsening of respiratory function, i.e., when patients had already been classified as group 2 patients (high-intensity group), this therapeutic measure did not affect their correct categorization.

### 2.3. Statistical Analysis

Continuous variables are expressed as ranges, means ± standard deviation, and medians. Variables were dichotomized using median (age, CTSS, P/F ratio, NLR, and LMR) or normal values (LDH, CRP, fibrinogen, AST, ALT, ALP, and albumin), as appropriate. The chi-square test was used for modeling the relationship between RS and other prognostic factors. Since the P/F ratio was used to monitor patient respiratory function and served as a benchmark to decide the optimal respiratory support, thus representing a strong confounding factor, it was removed from further analyses. Multivariate analysis with multiple logistic regression was used to individuate independent prognostic variables related to RS. The multicollinearity among variables supposed to have a high correlation was investigated with interaction analysis. The receiver operating characteristic (ROC) curve was applied to quantify the performance of different factors to predict the need for RS (no or mild/moderate RS vs. severe RS, group 1 vs. group 2, respectively) by computing the area under the curve (AUC), sensitivity, specificity, and positive predictive (+PV) and negative predictive (−PV) values. Particularly, +PV indicated the probability of respiratory failure when the specified factor was present; on the contrary, −PV indicated the probability of respiratory stationarity or improvement when the variable was absent. Thereafter, a comparison ROC curve among significantly independent factors was plotted. However, an ROC curve is unable to accurately quantify the effect over time of risk prediction offered by a prognostic variable. Thus, the analysis of the area under the curve (AUC), calculated using the time-dependent ROC curve for censored survival data, was carried out. It thus estimated the probability that, at a certain time point, a patient with a worsened pulmonary condition had been correctly predicted with respect to a patient who did not present with pulmonary impairment at that time. Higher AUC values indicated better predictive ability.

All analyses were two-sided, and *p* < 0.05 was considered to be statistically significant. Statistical analyses were carried out using the SPSS 21.0 software (SPSS Inc., Chicago, IL, USA) and the statistical package R (version 3.2.5, R Foundation for statistical computing) integrated with Medcalc^®^ software version 12.5.0.0 (Mariakerke, Belgium).

## 3. Results

One hundred twenty-three patients were eligible for the study. On admission, nearly 60% of patients (73 cases) were spontaneously breathing. Afterward, 78 patients (63.4%) were treated without the need for CPAP, NIV, and/or IOT, and could be all discharged; on the contrary, 45 patients (36.6%) needed CPAP, NIV, and/or IOT, with four deaths. Importantly, 36 patients who had been classified as group 1 patients on admission needed escalation of RS during their stay in the ward (switched from group 1 to group 2 patients in outcome evaluation), with two deaths. The patients’ characteristics are listed in Table 1.

**Figure 1:**
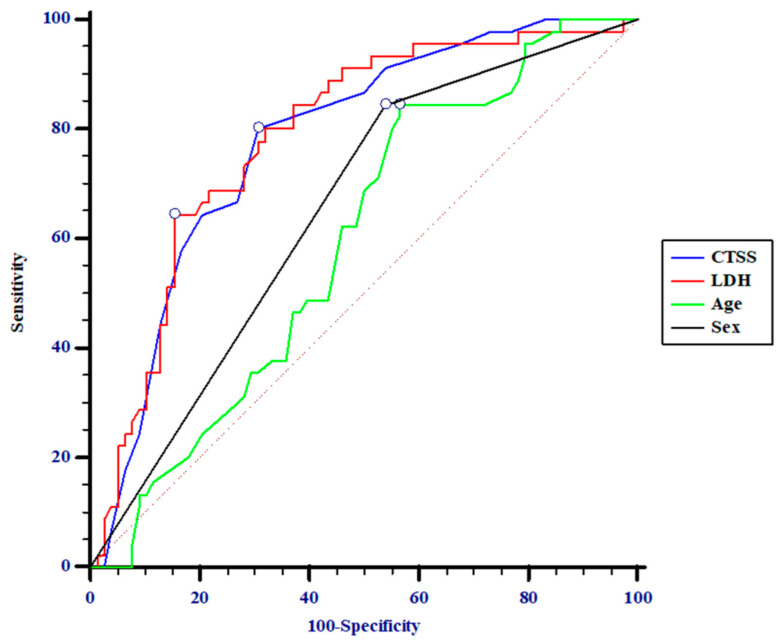
Comparison of ROC curves: CTSS–age, *p* = 0.0023; CTSS–gender, *p* = 0.0394; LDH–age, *p* = 0.0026; LDH–gender, *p* = 0.0360; age–gender, *p* = 0.3710; CTSS–LDH, *p* = 0.8724.

**Figure 2:**
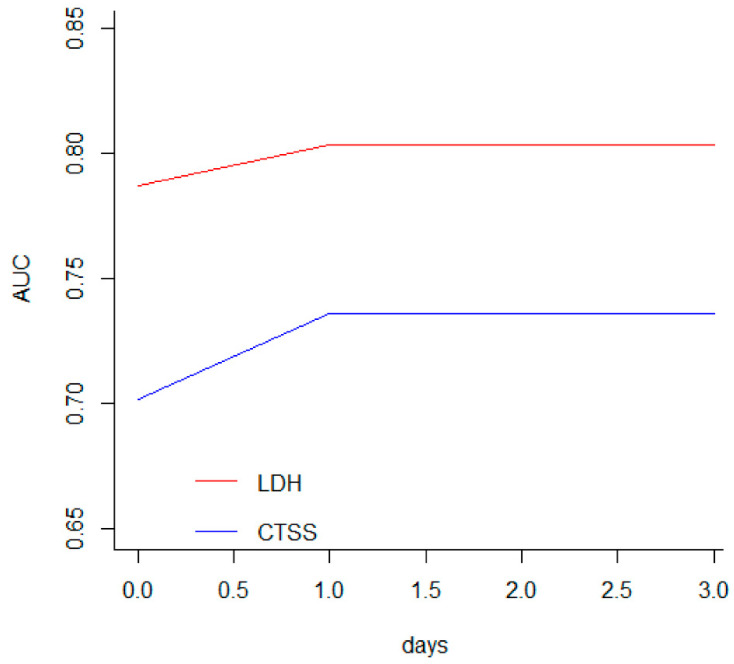
Analysis of the predictive accuracy of LDH and CTSS through the first 3 days from hospital admittance, computed by the time-dependent ROC analysis for censored survival data.

Overall, patients requiring a more aggressive RS had significantly higher CTSS and NLR scores with lower P/F ratios, as well as higher serum levels of LDH, CRP, D-dimer, and AST. After the multivariate logistic regression, a number of variables lost significance; only older age, male gender, and higher CTSS and LDH values were shown to be independent prognostic factors of a worse evolution of respiratory function (Table 2). The ROC curve confirmed the logistic regression results; in addition, the analysis provided corrected cut-off values correlated with the outcome. Men older than 56 years showing a CTSS of >7 and an LDH serum level of >328 U/L had the highest probability of requiring intensive RS (Table 3). Afterward, the most reliable predictive factors appeared to be CTSS and LDH. This latter parameter showed a +PV = 71%, meaning that 71% of patients with LDH >328 U/L had respiratory impairment, and a −PV = 81, meaning that 81% of patients with low LDH serum levels did not undergo aggressive treatment. Indeed, when analyzing the four variables together, age and gender were shown to perform worse than CTSS and LDH, with the latter showing the best AUCs, with no significant difference between their curves. By contrast, although the age- and gender-related curves were not different from each other, they performed significantly worse than CTSS and LDH (Figure 1). With regard to the correct prevision of outcome according to time, as evaluated by the time-dependent ROC curve for censored survival data, LDH was shown to perform better than CTSS throughout the study period. Interestingly, both curves rose on the first day and quickly reached a plateau that remained unchanged as the patients’ history continued, suggesting that the first hours were crucial in relation to pneumonia progression, with LDH values showing a very important role in predicting outcomes (Figure 2).

Ultimately, among the 60 patients with CTSS > 7 and thus believed to be at higher risk of high-intensity RS, 25 out of 33 patients (75%) with LDH > 328 U/L required indeed aggressive treatment. Accordingly, among the 27 patients with LDH values ≤ 328 U/L, 16 (59%) did not experience an escalation of respiratory support. In addition, among 63 patients with CTSS ≤ 7 believed to be at lower risk, 4 out of 8 patients with LDH > 328 U/L (50%) showed worsening respiratory function and underwent aggressive treatment. By contrast, among 55 patients with LDH values ≤ 328 U/L, only 5 (9%) experienced the need for high-intensity RS. This means that LDH correctly intercepted 16 (59%) and 4 (50%) patients over- or underestimated by CTSS (Table 4).

## 4. Discussion

This study provides a simple way to stratify COVID-19 patients on admission into two main groups, namely, patients requiring no RS or mild/moderate oxygen administration (i.e., by NC or HFNC, respectively) and patients requiring intensive treatment (from CPAP to OTI) for whom specialized pulmonary and intensivist care may be necessary. This method was denominated V:ERITAS, after the name and logo of our institution, to indicate the ability to predict the spreading of lung inflammation.

The first implication of the study is the identification of a subgroup of patients that can be properly cared for in general internal medicine wards dedicated to COVID-19 patients. As COVID-19 pneumonia occurrence and severity cannot be precisely predicted on admission if not yet full-blown, an important issue may be whether the expected level of care may be appropriate for a given patient in an internal medicine ward. Further complicating things, during pandemic peaks, a surge of COVID-19 patients in need of RS may be expected to congest even low-intensity wards, compelling non-intensivist doctors to develop competence and skills in order to manage critically ill patients as well [15]. The prediction of the respiratory outcome may allow early decisions as to which patients can be conservatively and safely managed in a lower intensity setting (e.g., the internal medicine ward), while more appropriately addressing high-risk patients to intensivist care in case of prediction of high-intensity RS (from CPAP on); furthermore, the time saved before the worsening of COVID-19 pneumonia may be lifesaving, as some patients may experience rapidly evolving respiratory distress [16]. The second implication is that intensive care unit beds, whose availability is crucial during pandemic peaks to warrant each patient all the required levels of care [17], may be better preserved for those predicted to need intensive RS. For instance, patients predicted to need mild/moderate RS (i.e., up to HFNC) may be retained in internal medicine wards, thus avoiding depleting beds in intensivist units.

In our experience, it is important to note that 36 patients evolved from the low-intensity to the high-intensity level of care during their hospital stay, two of them ultimately dying of COVID-19 pneumonia. All these patients were cared for in the internal medicine ward (only two of them were eventually transferred to the intensivist ward), with attending physicians urged to gain quick knowledge of intensive care procedures. Prediction of the worse outcome on admission might have aided the optimization of overall management by favoring sooner-than-planned aggressive therapeutic decisions to prevent widespread lung damage (e.g., early use of anti-cytokine therapies), early notification to the intensivist personnel of their impending involvement, and mitigation of physician burnout.

The proposed method is easy to use, as only two main variables are taken into account, and it does not require elaborate calculations. By simply obtaining a serum LDH value and the CTSS, which are routinely evaluated in COVID-19 patients on admission, the clinician may immediately allocate the patient in the low/moderate or high-risk category and, consequently, decide to conservatively manage the patient or not. In this regard, our approach differs from others that have been recently proposed. Indeed, these latter approaches require the consideration of multiple variables and, sometimes, because of their complexity, even ad hoc calculators [7,8,9,18,19,20]. Another advantage is represented by the fact that the predictive ability is independent of patient comorbidities, which instead need to be taken into account in other previously proposed risk models [7,8,9,18,19,20]. For instance, the BUSTO COVID-19 score takes into account eight clinical and laboratory variables to stratify patients into four groups with an increasing risk of death and other adverse medical outcomes (admission to the ICU and mechanical ventilation), but to quickly calculate the score, a web-based calculator is needed [7]; moreover, a lung ultrasound assessment is required to finalize the score. The SIMI score is easier to obtain but still requires the assessment of six variables independently scored using pre-defined cut-off values [8]. Further complexity characterizes the COVID risk score constructed on 10 variables, including several comorbidities, which again requires an online calculator to yield the likelihood of a hospitalized patient with COVID-19 developing critical illness [9]. Finally, a risk prediction model for assessment of COVID-19 pneumonia progression, developed from a cohort of patients from Wuhan, China, again entails the evaluation of multiple (nine) variables, including several comorbidities [18]. Although all of the above-mentioned risk scores show somewhat similar performance, they nonetheless lack ease of use, which, on the contrary, characterizes our method, along with good reliability.

From a pathophysiologic point of view, the two variables identified in this study are reciprocally intertwined in the predictive function that emerged from the statistical analysis. Specifically, CTSS is a semiquantitative measure to grade the extent (i.e., the area) of lung involvement [10]; however, it cannot give an estimate of parenchymal lung tissue damage severity. This is exemplified by the findings of patients with the same CTSS but different outcomes, despite matching of characteristics and comorbidities. On the other hand, LDH serum levels may reflect the severity of lung tissue damage [21], regardless of the area of lung involvement as measured by CTSS. Thus, by combining the two variables, a better definition of pulmonary involvement in terms of overall damage severity may be obtained. An immediate consequence is whether the use of lung LDH isoenzyme serum levels may improve the predictive ability of our risk score with respect to total LDH serum levels [21].

Clearly, this study has some limitations. First, because of the relatively small patient population, our results need to be confirmed in larger cohorts; second, the study was conducted in a single center, and therefore, validation in different geographic areas would be desirable; finally, because of the retrospective nature of the study, potential bias may have not been recognized. Nevertheless, its ease of use may justify prospective validation in large cohorts of patients.

## 5. Conclusions

Two easy-to-obtain parameters (namely, CTSS and LDH) were able to reliably predict a worse evolution of COVID-19 pneumonia with values of >7 and >328 U/L, respectively in patients admitted to an internal medicine ward. The potential impact on the optimization of patient management strategies needs to be investigated in prospective studies.

## Figures and Tables

**Table 1:** Clinicopathological characteristics and respiratory support outcome.

Respiratory Support Outcome				
	No.	No Respiratory Support, NC, or HFNC	Need for CPAP, NIV, or OTI	*p* *
Age (years) ^†^				0.7036
mean 62 ± 4				
range 26–89				
≤63	67	44	23	
>63	56	34	22	
Gender				0.0012 ^‡^
Male	80	42	38	
Female	43	36	7	
CTSS ^†^				<0.0001 ^‡^
mean 7.7 ± 4.4				
range 0–17				
≤7	63	54	9	
>7	60	24	36	
P/F ratio ^†^				<0.0001 ^‡^
mean 260 ± 101				
range 69–500				
>268	61	57	4	
<268	63	21	41	
LDH (U/L) ^a^				<0.0001 ^‡^
mean 297 ± 114				
range 125–243				
≤243	48	43	5	
>243	75	35	40	
NLR ^†^				0.0002 ^‡^
mean 8.1 ± 6.8				
range 1.0–47.8				
≤6.2	61	49	12	
>6.2	62	29	33	
LMR ^†^				0.0523
mean 2.2 ± 1.2				
range 0.6–7.0				
>1.9	62	45	17	
<1.9	61	33	28	
CRP ^a^				0.0305 ^‡^
mean 52 ± 54				
range 0.09–252				
≤5	18	16	2	
>5	105	62	43	
Fibrinogen (mg/dL) ^a^				0.4444
mean 590 ± 193				
range 208–1000				
≤3.75	13	10	3	
>3.75	110	68	42	
D-dimer (ng/mL) ^a^				0.0445 ^‡^
mean 418 ± 528				
range 74–4662				
≤260	57	42	15	
>260	66	36	30	
AST (U/L) ^a^				0.0004 ^‡^
mean 39 ± 42				
range 9–356				
≤33	78	59	19	
>33	45	19	26	
ALT (U/L) ^a^				0.5175
mean 46 ± 42				
range 3–221				
≤49	85	56	29	
>49	38	22	16	
ALP (U/L) ^a^				0.7459
mean 46 ± 42				
range 3–221				
≤49	115	73	42	
>49	8	5	3	
Albumin (g/dL) ^a^				0.9261
mean 3.8 ± 0.4				
range 2.7–4.8				
≥3.5	103	66	37	
<3.5	20	12	8	

CPAP, continuous positive airway pressure; HFNC, high-flow nasal cannula; NC, nasal cannula; NIV, non-invasive ventilation; OTI, oro-tracheal intubation; CTSS, (chest) computed tomography severity score; P/F, PaO_2/_FiO_2_; LDH, lactate dehydrogenase (normal range: 120–243 U/L); NLR, neutrophil to lymphocyte ratio; LMR, lymphocyte to monocyte ratio; CRP, C-reactive protein (normal range: 0–5); AST, aspartate aminotransferase (normal range: 5–33 U/L); ALT, alanine aminotransferase (normal range: 5–49 U/L); ALP, alkaline phosphatase (normal range: 50–106 U/L); * χ^2^ test; ^†^ age, CTSS, P/F ratio, NLR, and LMR were dichotomized by median values; ^‡^ significant value; ^a^ LDH, CRP, fibrinogen, AST, ALT, ALP, and albumin were dichotomized by normal values.

**Table 2:** Multivariate analysis with logistic regression.

Variable	Coefficient	Standard Error	*p*
Age	0.054682	0.024616	0.0263
Gender	−1.37100	0.62439	0.0281
CTSS	0.14322	0.075131	0.0566
LDH	0.0059958	0.0034632	0.0834
NLR	0.057745	0.054739	0.2915
LMR	−0.024549	0.24068	0.9188
CRP	0.0088529	0.0065703	0.1778
Fibrinogen	0.00084544	0.0016936	0.6176
D-dimer	0.00023585	0.00054009	0.6623
AST	−0.010337	0.0096558	0.2844
ALT	0.0051031	0.0070821	0.4712
ALP	0.0027839	0.0038827	0.4734
Albumin	0.20774	0.70751	0.7691

CTSS, (chest) computed tomography severity score; LDH, lactate dehydrogenase; NLR, neutrophil to lymphocyte ratio; LMR, lymphocyte to monocyte ratio; CRP, C-reactive protein; AST, aspartate aminotransferase; ALT, alanine aminotransferase; ALP, alkaline phosphatase.

**Table 3:** Performance of different variables in the prediction of respiratory support.

Variable	AUC	*p*	Sensitivity %	Specificity %	+PV %	−PV %
Age > 56 years	0.59 (0.5–0.7)	0.0678	84 (70–93)	44 (32–55)	46 (35–58)	83 (68–93)
Male gender	0.65 (0.6–0.7)	0.0021	84 (70–93)	46 (35–58)	47 (36–59)	84 (69–93)
CTSS > 7	0.78 (0.7–0.8)	<0.0001	80 (65–90)	69 (58–79)	60 (46–72)	86 (75–93)
LDH > 328 U/L	0.78 (0.7–0.8)	<0.0001	64 (49–78)	84 (75–92)	71 (54–84)	81 (70–88)

The performance was computed using the receiver operating characteristics (ROC) curve analysis. AUC, area under the ROC curve; +PV, positive predictive value; −PV, negative predictive value; CTSS, (chest) computed tomography severity score; LDH, lactate dehydrogenase. Values in parentheses indicate 95% confidence interval.

**Table 4:** Comparison of respiratory support prediction by CTSS and LDH.

	LDH ≤ 328 U/L 82 Patients	LDH > 328 U/L 41 Patients
**CTSS ≤ 7** 63 patients	55 (5)	8 (4)
**CTSS > 7** 60 patients	27 (11)	33 (25)

Numbers in parentheses indicate number of patients undergoing intensive respiratory support. CTSS, (chest) computed tomography severity score; LDH, lactate dehydrogenase.

## Data Availability

The datasets analyzed during the current study are available from the corresponding author upon reasonable request.
